# Optimization of a Simulated Annealing Algorithm for S-Boxes Generating

**DOI:** 10.3390/s22166073

**Published:** 2022-08-14

**Authors:** Alexandr Kuznetsov, Lukasz Wieclaw, Nikolay Poluyanenko, Lukasz Hamera, Sergey Kandiy, Yelyzaveta Lohachova

**Affiliations:** 1 Department of Political Sciences, Communication and International Relations, University of Macerata, Via Crescimbeni, 30/32, 62100 Macerata, Italy; 2 Department of Information and Communication Systems Security, Faculty of Comupter Science, V. N. Karazin Kharkiv National University, 4 Svobody Sq., 61022 Kharkiv, Ukraine; 3 Department of Information Systems and Technologies Security, JSC “Institute of Information Technologies”, Bakulin St., 12, 61166 Kharkiv, Ukraine; 4 Department of Computer Science and Automatics, Faculty of Mechanical Engineering and Computer Science, University of Bielsko-Biala, Willowa 2, 43-300 Bielsko-Biala, Poland

**Keywords:** simulated annealing algorithm, nonlinear substitutions, iterative search, computational complexity, S-box

## Abstract

Cryptographic algorithms are used to ensure confidentiality, integrity and authenticity of data in information systems. One of the important areas of modern cryptography is that of symmetric key ciphers. They convert the input plaintext into ciphertext, representing it as a random sequence of characters. S-boxes are designed to complicate the input–output relationship of the cipher. In other words, S-boxes introduce nonlinearity into the encryption process, complicating the use of different methods of cryptanalysis (linear, differential, statistical, correlation, etc.). In addition, S-boxes must be random. This property means that nonlinear substitution cannot be represented as simple algebraic constructions. Random S-boxes are designed to protect against algebraic methods of cryptanalysis. Thus, generation of random S-boxes is an important area of research directly related to the design of modern cryptographically strong symmetric ciphers. This problem has been solved in many related works, including some using the simulated annealing (SA) algorithm. Some works managed to generate 8-bit bijective S-boxes with a nonlinearity index of 104. However, this required enormous computational resources. This paper presents the results of our optimization of SA via various parameters. We were able to significantly reduce the computational complexity of substitution generation with SA. In addition, we also significantly increased the probability of generating the target S-boxes with a nonlinearity score of 104.

## 1. Introduction

Data encryption algorithms with symmetric keys are used in modern computer systems to ensure confidentiality, integrity, authenticity and other information security services [1,2,3]. The main condition for using such encryption algorithms is the availability of a secret key, which is identical for the sender and receiver of data.

An important component of most modern secret-key ciphers is nonlinear substitution (S-boxes). These boxes are designed to introduce complex nonlinear relationships into the plaintext–ciphertext relationship. In fact, S-boxes play a crucial role in providing cryptographic strength. Using classical terms of the theory of secret systems [4], S-boxes provide the confusion property, which plays a crucial role in protecting against differential, linear, statistical, correlation and many other types of cryptanalysis [1,5].

According to modern concepts, nonlinear substitutions in cryptography should be random [2]. Nonrandom methods of substitution generation can cause vulnerabilities in cryptoalgorithms [6,7]. For example, the well-known encryption algorithm AES [8] uses algebraic (not random) methods of S-box generation, and this fact was the reason for the appearance of algebraic cryptanalysis [9,10]. The simplicity of algebraic construction of S-box cipher AES is used to criticize this cryptoalgorithm. Thus, methods of generating S-boxes should be based on the use of random substitution processes.

One of the well-known methods for generating S-boxes is the simulated annealing (SA) algorithm [11,12,13,14]. The name of this algorithm comes from annealing in metallurgy, when heating and controlled cooling of a metal determine its physical properties [15]. These processes are simulated by a computer program for nonlinear substitution generation. The initial temperature sets the probability of random change in the S-box. This temperature gradually decreases, which leads to a gradual reduction in random changes. As a result, the process solidifies, and we have a final stand, the cryptographic properties of which are determined by the SA parameters.

It should be noted that SA suffers from the capture of local optima [15,16]. Nevertheless, this algorithm is well suited for solving the substitution generation problem. The global optimum in this problem corresponds to the maximum nonlinearity of the substitution, and this is achieved by using special algebraic structures in the finite field. For example, known algebraic constructs from [17,18] provide maximum nonlinearity of Boolean mappings (these very constructs were used to generate AES cipher substitutions [8]). Such mathematical constructs are described by simple algebraic equations, which can potentially be used to find cipher vulnerabilities [6,7,9]. We are interested in generating random highly nonlinear S-boxes that have no hidden mathematical structures, i.e., we are looking for local optimums of nonlinearity, and SA is well suited for this problem.

Substitution generation using SA has been investigated by many authors. However, the computational complexity of the known solutions is very high. For example, it took more than 3 million iterations to generate an 8-bit bijective substitution with nonlinearity 104 in [12]. In addition, the probability of generating a target S-box is also very low. For example, in [11,19], the probability of generating a statement with nonlinearity 102 was about 0.5%.

In this paper, we optimized the SA parameters to generate target S-boxes (hereinafter, the target refers to an 8-bit bijective substitution with nonlinearity 104). Our optimization of SA allowed us to significantly reduce the computational complexity (about 450 thousand iterations are required) and increase the probability (more than 50%) of generating the target S-boxes.

## 2. Related Work

Evolutionary techniques of computational intelligence are used to solve complex computational problems related to mathematical optimization and search for the best element by some criterion from some set of available alternatives [20,21]. Evolutionary algorithms are used to solve various combinatorial optimization problems [22,23]. For example, these include global and engineering optimization problems [24], production re-planning in Industry 4.0 [25], optimization [26] and routing problems [27], and industrial production optimization [28].

One of the most efficient methods for solving global optimization problems (especially discrete and combinatorial optimization) is SA. This algorithm is inspired by the natural processes that occur in the annealing of metals. The algorithm is based on the simulation of the physical process that occurs when a substance crystallizes. It is assumed that the atoms of matter are almost lined up in a crystal lattice, but transitions of individual atoms from one cell to another are still allowed. The higher the temperature, the greater the activity of the atoms. The temperature is gradually lowered, which leads to a decrease in the probability of transitions. A stable crystal lattice corresponds to the minimum energy of the atoms. In computational intelligence, this process is simulated as a computational algorithm for solving a global optimization problem, i.e., it is necessary to find the point (set of points) where the minimum (or maximum) of some target function is reached.

The first works that used SA for the problem of generating nonlinear S-boxes were the works of John A. Clark [11,19]. The author managed to generate an 8-bit substitution with a nonlinearity of 102. In addition, he proposed a cost function for SA, which was used in further related works [29,30,31,32]. In [30,32,33], other forms of the cost function were investigated. In [12,13,14,34], SA for generation of highly nonlinear S-boxes was investigated. However, the computational complexity of solving this problem turned out to be very high. For example, in [12], they managed to generate an 8-bit bijective S-box with nonlinearity 104, but it required more than 3 million iterations. In [13], the authors managed to generate a substitution with nonlinearity 100, which is significantly lower than other known results. SA was also used in [14] to generate permutations, but only nonlinearity 92 was achieved.

Thus, SA is used to generate nonlinear substitutions in cryptography. However, the computational complexity of the generation algorithm is very high. In addition, the probability of generating a target S-box is very low. For example, in [11,19], the probability of generating a statement with nonlinearity 102 was about 0.5%.

## 3. Materials and Methods

The main characteristic of heuristic search is the cost function C(S), which displays the state of the system in some natural way.

We used the function from [33,35] as the substitution cost function:

(1) $$C(S)=\sum_{i=1}^{255}{\left|\left|\max\left(WHT\right)\right|-X\right|}^{R}.$$

where:

▪ WHT—Walsh–Hadamard spectral coefficients;
▪ X and R—some parameters of the target function WHS.
▪ As the optimal parameters of the function (1) selected [33,35]:
▪ X=36 as the maximum permissible value, which reduces C(S), but does not lead to a significant effect on its adequate relationship with the nonlinearity of the S-box;
▪ R=4 as the maximum allowable value, increasing the range of function values C(S), which can improve the “sensitivity” of S-box formation algorithms.

Note that when calculating the cost function C(S), the nonlinearity of the S-box was simultaneously calculated:

(2) $$N_{f}=\frac{1}{2}\cdot \left(2^{n}-\max\left(WHT\right)\right)=128-\frac{1}{2}\cdot \max\left(WHT\right).$$

The main advantage of SA is its ability to escape from local optima. This is achieved due to the ability of the algorithm to take some deteriorating steps in the local understanding but ensure that the algorithm advances and finds a better state.

The first application of the simulated annealing algorithm to the problem of S-box generation was given in [8]. At the beginning of the algorithm, the initial solution $S_{best\_sbox}$, which provides, firstly, the property of bijectivity of the S-block and, secondly, its random nature, is formed. Then, a slight modification of the current state is performed. The new S-block state will be denoted as $S_{n}$.

After each modification, the cost Function (1) is calculated for $S_{n}$. This value is compared with the previous best solution, i.e., with the value of the cost function for $S_{best\_sbox}$. If Condition (3) holds,

(3) $$C(S_{n})\leq C(S_{best\_sbox}),$$

then the algorithm takes $S_{best\_sbox}=S_{n}$. Using Condition (3) increases the number of possible solutions.

The main advantage of SA is the possibility of making a worsening decision, i.e., one that does not satisfy Condition (3). In our algorithm, if Condition (3) is not satisfied, the algorithm makes a worsening decision $S_{best\_sbox}=S_{n}$ with Probability (4):

(4) $$\mathrm{Pr}\left(S_{best\_sbox}=S_{n}\right)=e^{\left(\frac{C(S_{best\_sbox})-C(S_{n})}{T_{i}}\right)},$$

where $T_{i}=\alpha \cdot T_{i-1}$ is the temperature equivalent in the process of metal annealing. In our case, this is a parameter characterizing the probability of deterioration of the current state.

**Figure 1:**
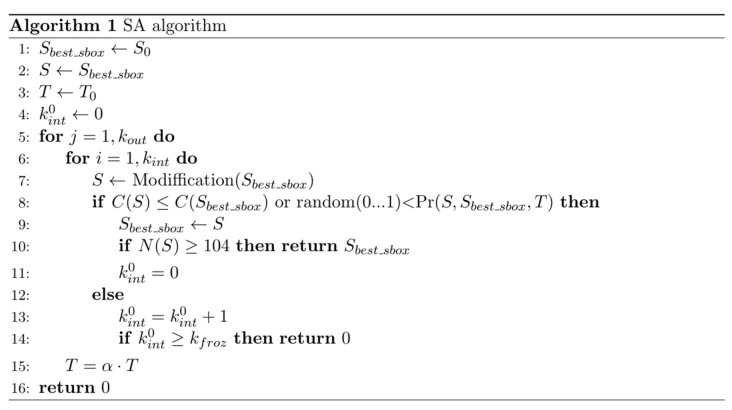
Pseudocode of the proposed annealing simulation algorithm.

The pseudocode of the implemented SA is shown in Figure 1.

At each value of the current temperature $T_{i}$, the algorithm performs $k_{int}$ iterations (let us call them inner cycles). The number of changes in the current temperature is determined by the parameter $k_{out}$ (let us call it the number of external cycles). In order to limit the number of external iterations that do not yield improvements, we also introduced the parameter $k_{froz}$—the maximum number of external iterations without improvements.

The implemented algorithm of S-boxes generation was adapted to run in multi-threaded search mode.

## 4. Test Cases

When implementing the simulated annealing algorithm for S-box generation, we used the following initial parameters:

▪ $K_{THREAD}$—the number of threads in which the simultaneous search takes place. In our case, $K_{THREAD}$ = 30, which corresponded to the maximum number of threads supported by the computer’s processor;
▪ $T_{0}$—initial “temperature” value. It is stated in [36] that $T_{0}$ should provide an initial worst-case decision probability of 50–80%. We investigated the search efficiency at different values of $T_{0}$;
▪ α—“cooling coefficient”, which determines how much the temperature decreases at each iteration of the algorithm. We investigated the search efficiency at different values of α;
▪ $k_{int}$—parameter, which specifies the number of internal cycles that the local search algorithm can perform at each temperature. We applied $k_{int}$ = 650 (i.e., the total number of internal tests was 30×650=19,500); Stopping criteria. The stopping criteria used were as follows:
  – $N_{f}$—the target value of the nonlinearity (4) of the S-box. In our experiments, we limited ourselves to the value $N_{f}=104$, i.e., the search stops when $S_{n}$ with nonlinearity 104 is found;
  – $k_{out}$—the maximum number of external cycles, i.e., how many times the SA algorithm was allowed to lower the temperature and continue searching before it stopped. We used $k_{out}$ = 50;
  – $k_{froz}$—the number of consecutive outer cycles in which no improvement of the cost function was found. We used $k_{froz}$ = 5.

The individual parameters of the algorithm ($k_{int}$, $k_{out}$, $k_{froz}$) were chosen from the considerations given in [35].

The initial temperature varied from a value where the probability of making the worst decision was almost zero to a higher one where the probability was close to one.

The increase in $T_{0}$ was performed according to the rule:

(5) $$T_{0}^{i+1}=1.13\cdot T_{0}^{i}.$$

For each $T_{0}^{i}$ of (5), 100 runs of the simulated annealing algorithm were performed.

The parameter α varied from 0.6 to 0.95:

▪ for α=0.6, 10,100 runs of the search algorithm were performed;
▪ for α=0.7, 7600 launches were performed;
▪ for α=0.8, 5700 launches were performed;
▪ for α=0.9, 8200 launches were performed;
▪ for α=0.95, 8200 launches were performed.

The constraint $k_{froz}$ = 5 resulted in the loss of some solutions for which the algorithm could still find the target S-box with nonlinearity 104. However, the expediency of further search was considered small compared to the time spent.

## 5. Results

The first part of the experiments consisted in estimating the number of runs of the search algorithm for which no improvement of the cost function was found for a long time. The obtained results are shown in Table 1.

As we can see from the data in Table 1, there is an increase in time with increasing α in which no improvement in the cost function was found. This can be explained by an increase in the share of accepted value function deteriorations, which in turn leads to an increase in the probability of exiting the local minimum.

**Figure 2:**
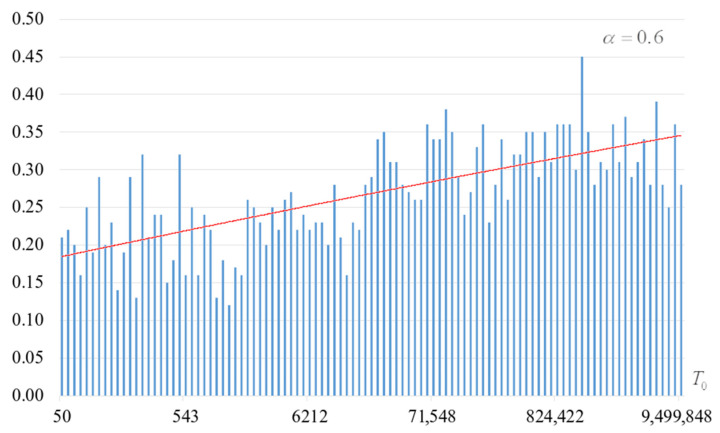
The probability of forming the target S-box at α=0.6.

**Figure 3:**
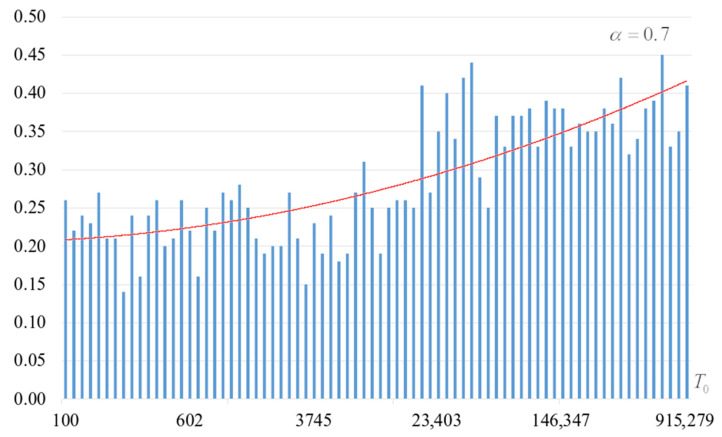
The probability of forming the target S-box at α=0.7.

**Figure 4:**
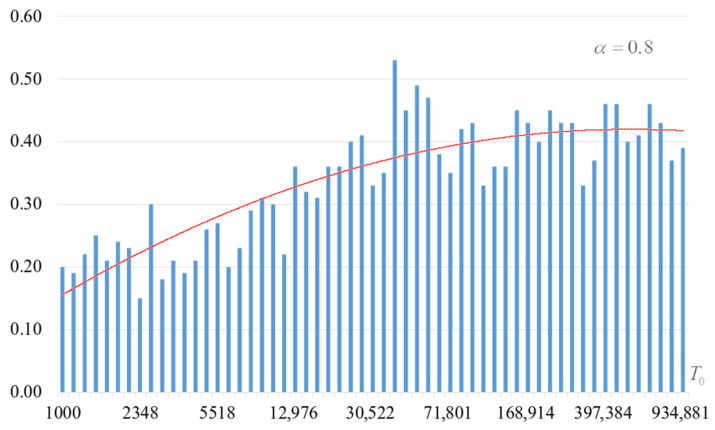
The probability of forming the target S-box at α=0.8.

**Figure 5:**
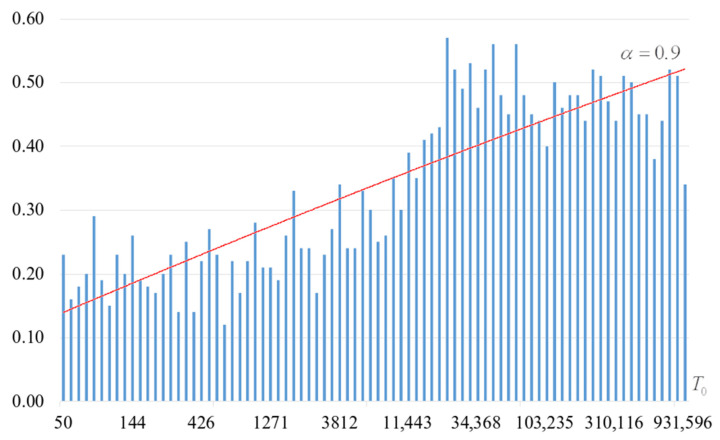
The probability of forming the target S-box at α=0.9.

**Figure 6:**
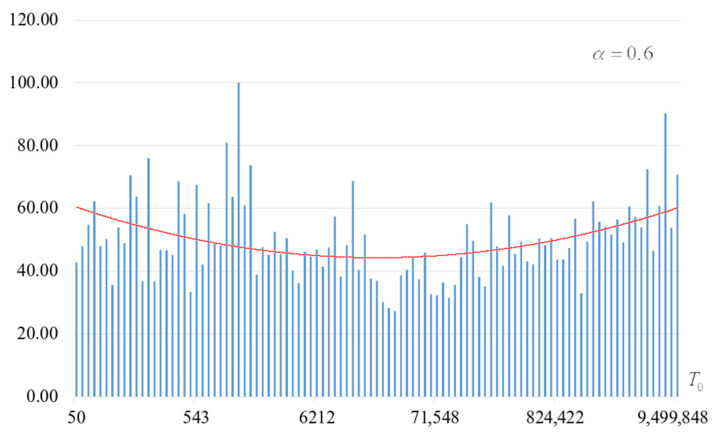
Average time (s) of target S-box formation at α=0.6.

**Figure 7:**
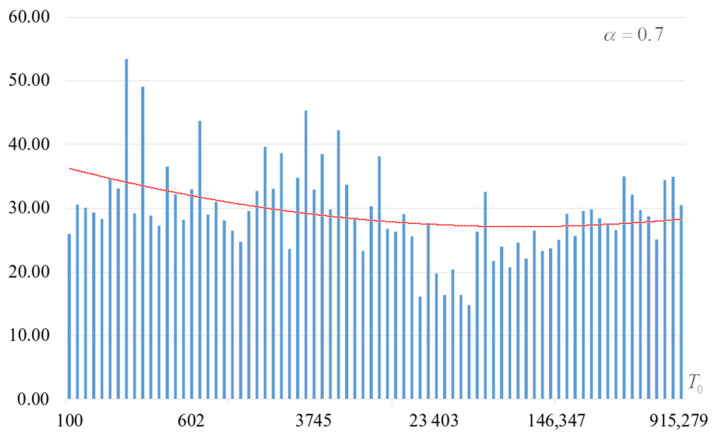
Average time (s) of target S-box formation at α=0.7.

**Figure 8:**
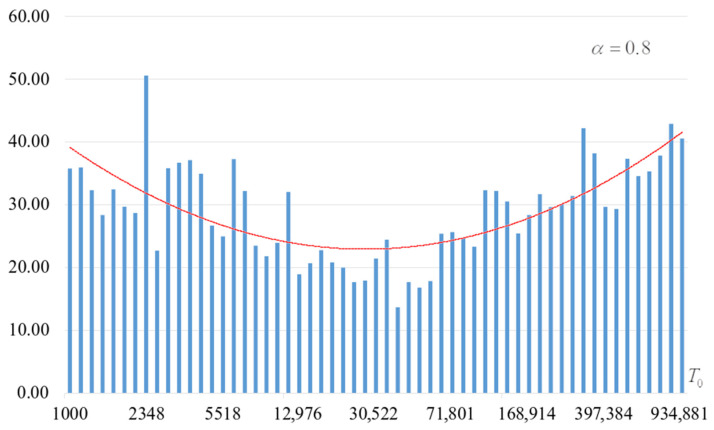
Average time (s) of target S-box formation at α=0.8.

**Figure 9:**
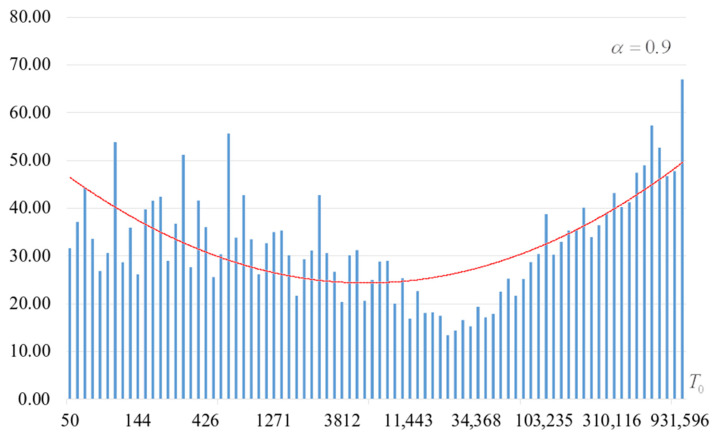
Average time (s) of target S-box formation at α=0.9.

The second part of the experiments consisted in estimating the probability of forming the target S-box. The results are shown in Figure 2, Figure 3, Figure 4 and Figure 5. The probabilities were estimated as the ratio of the number of generated target S-boxes to the total number of runs of the algorithm. Additionally, we measured the average time to generate substitutions. The results obtained are shown in Figure 6, Figure 7, Figure 8 and Figure 9. The average generation time includes the time spent on unsuccessful runs of the search algorithm. For all graphs in Figure 2, Figure 3, Figure 4, Figure 5, Figure 6, Figure 7, Figure 8 and Figure 9, we additionally present the trend line.

**Figure 10:**
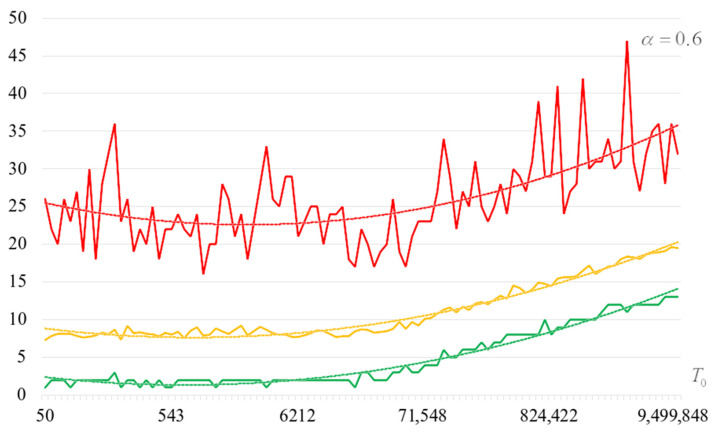
The dependence of the number of iterations of an external loop (until one of the criteria for stopping the algorithm is met), α=0.6.

**Figure 11:**
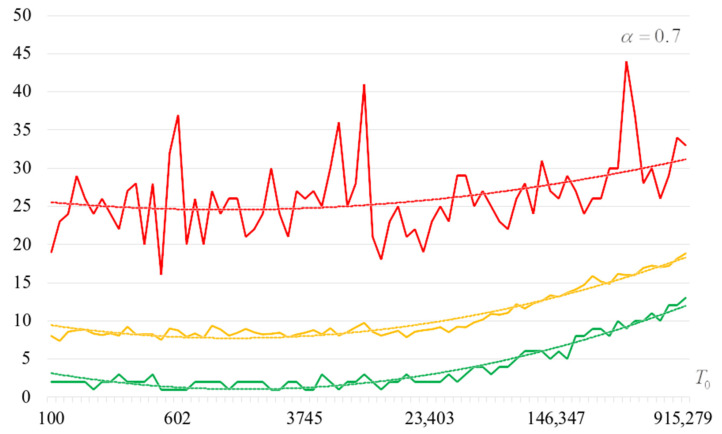
The dependence of the number of iterations of an external loop (until one of the criteria for stopping the algorithm is met), α=0.7.

**Figure 12:**
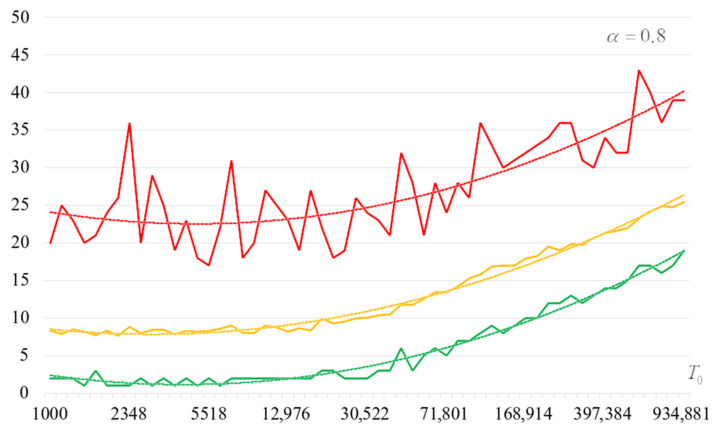
The dependence of the number of iterations of an external loop (until one of the criteria for stopping the algorithm is met), α=0.8.

**Figure 13:**
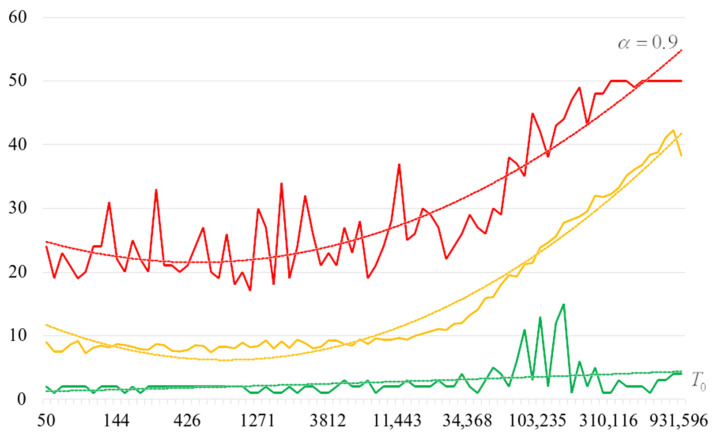
The dependence of the number of iterations of an external loop (until one of the criteria for stopping the algorithm is met), α=0.9.

To detail the obtained results, Figure 10, Figure 11, Figure 12 and Figure 13 show the dependencies of the number of iterations of the outer loop (until one of the criteria for stopping the algorithm is fulfilled):

- The upper curve (red) corresponds to the maximum number of iterations;
- The middle curve (yellow) corresponds to the average number of iterations;
- The lower curve (green) corresponds to the minimum number of iterations.

**Figure 14:**
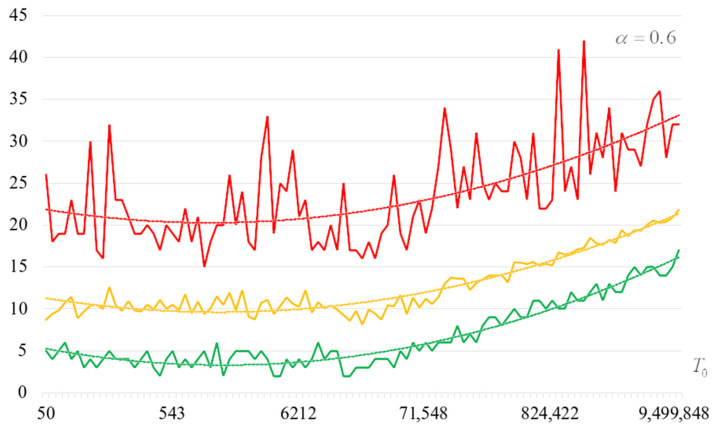
The dependencies of the number of iterations of the outer loop (until one of the criteria for stopping the algorithm is fulfilled) if the algorithm is successfully started, α=0.6.

**Figure 15:**
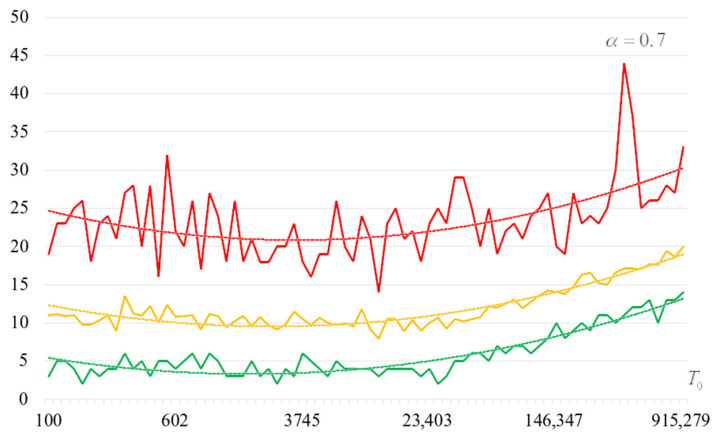
The dependencies of the number of iterations of the outer loop (until one of the criteria for stopping the algorithm is fulfilled) if the algorithm is successfully started, α=0.7.

**Figure 16:**
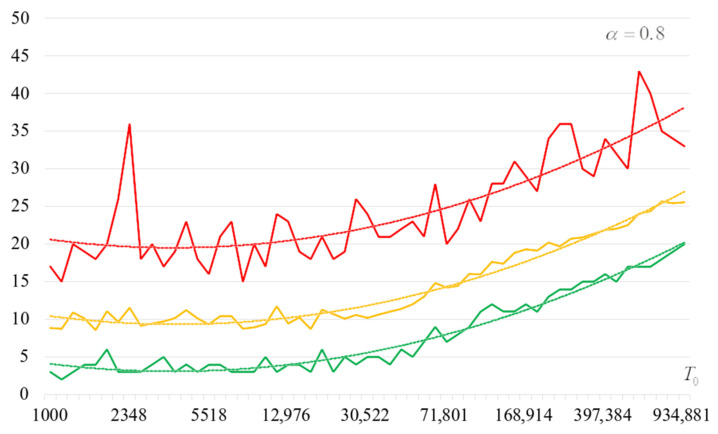
The dependencies of the number of iterations of the outer loop (until one of the criteria for stopping the algorithm is fulfilled) if the algorithm is successfully started, α=0.8.

**Figure 17:**
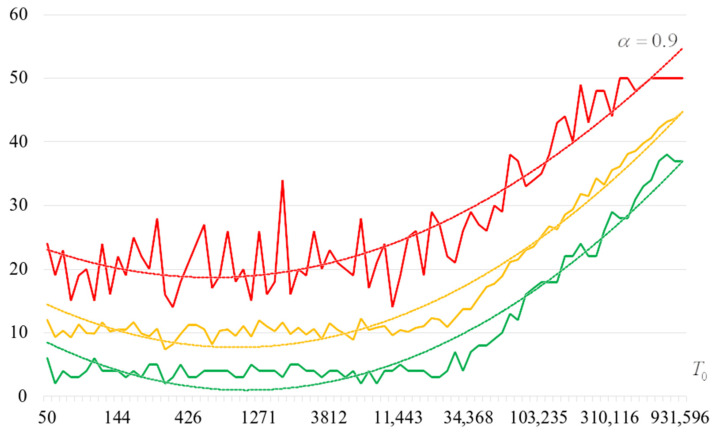
The dependencies of the number of iterations of the outer loop (until one of the criteria for stopping the algorithm is fulfilled) if the algorithm is successfully started, α=0.9.

The most interesting dependencies are shown in Figure 14, Figure 15, Figure 16 and Figure 17. These dependencies are dependences of number of iterations of external loop (until one of the criteria of algorithm stopping is fulfilled) under the condition of successful start of the algorithm. In other words, dependencies in Figure 14, Figure 15, Figure 16 and Figure 17 correspond to the cases when running the search algorithm resulted in generation of the target S-box:

- The upper curve (red) corresponds to the maximum number of iterations;
- The middle curve (yellow) corresponds to the average number of iterations;
- The lower curve (green) corresponds to the minimum number of iterations.

Analysis of the dependencies shown in Figure 2, Figure 3, Figure 4 and Figure 5 shows that when the initial temperature $T_{0}$ increases, the probability of forming the target S-box also increases. However, the average generation time does not decrease. This can be clearly seen in Figure 6, Figure 7, Figure 8 and Figure 9. For each value of α, we have the “optimal” value of the initial temperature $T_{0}$, at which the generation time is minimized. This conclusion is also confirmed by Figure 10, Figure 11, Figure 12, Figure 13, Figure 14, Figure 15, Figure 16 and Figure 17, where we see that all iteration number curves can be optimized by the value of the initial temperature $T_{0}$.

**Figure 18:**
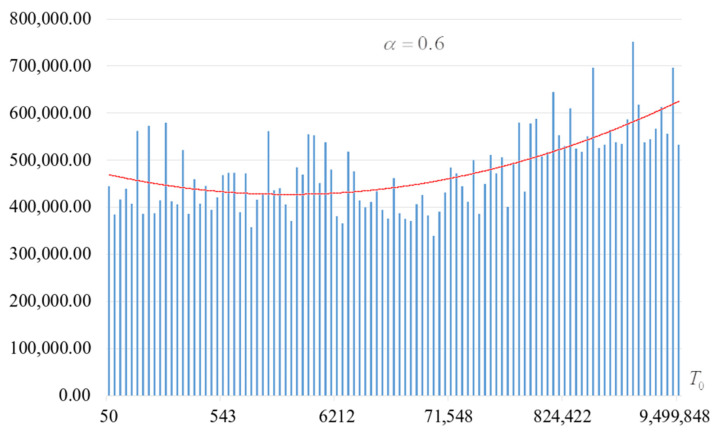
The summary number of the arithmetic mean number of iterations when searching for the target S-box, α=0.6.

**Figure 19:**
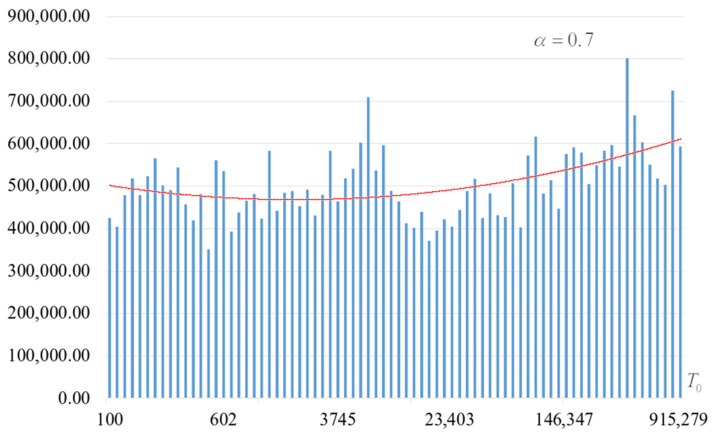
The summary number of the arithmetic mean number of iterations when searching for the target S-box, α=0.7.

**Figure 20:**
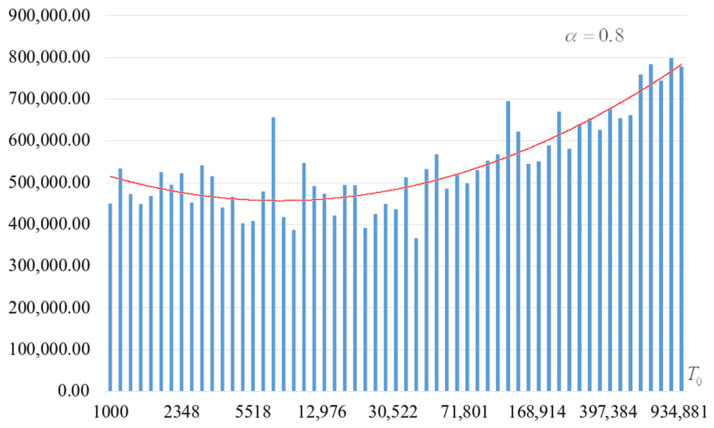
The summary number of the arithmetic mean number of iterations when searching for the target S-box, α=0.8.

**Figure 21:**
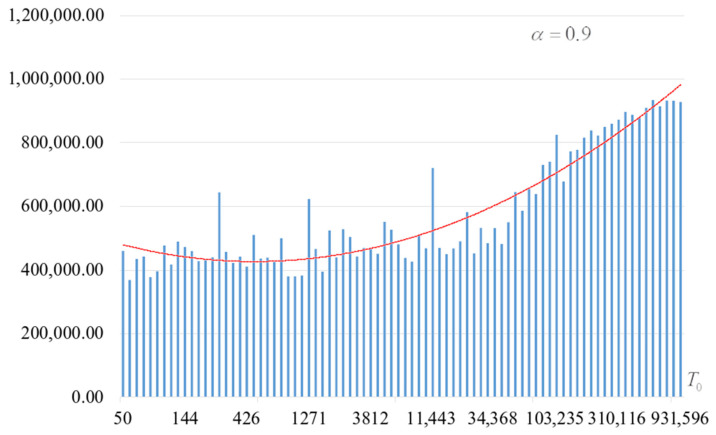
The summary number of the arithmetic mean number of iterations when searching for the target S-box, α=0.9.

The final dependencies of the total number of iterations (external and internal cycle) are shown in Figure 18, Figure 19, Figure 20 and Figure 21.

The analysis of the dependencies shown in Figure 18, Figure 19, Figure 20 and Figure 21 allows us to draw the following conclusions:

- As the value of $T_{0}$ grows, the total number of iterations is almost the same until a certain value ($T_{0}=30,000\ldots 70,000$, depending on α) and then begins to grow rapidly, which causes a significant increase in the time to execute each run of the algorithm.
- If the α is increased, the total number of search iterations decreases, i.e., substitution generation is performed faster.

## 6. Discussion

At small values of the initial temperature, the probability of making a worsening decision is very small, and therefore the simulated annealing algorithm behaves like a normal algorithm for finding a local minimum and, accordingly, has the same probability value of forming the target S-box and the average search time.

As the initial temperature increases, the probability of making worsening decisions increases, leading to an exit from the current state, which on the one hand may be an unnecessary local minimum and on the other hand may be one of the acceptable decisions that can lead to the formation of the target S-box. Analysis of the results indicates that the arithmetic mean value of the nonlinearity $N_{f}$ is reached approximately at the outer loop iteration, which corresponds to the current temperature of the found minimum ($T_{0}=$20,000 … 40,000).

A higher temperature leads to so-called *non-productive deteriorations*, i.e., deteriorations that lead to a permanent rollback of the found solution to the deteriorated state. Therefore, more iterations that are performed under nonproductive deteriorations can also be referred to *nonproductive iterations*, i.e., those that do not lead to improvement of the overall state of the system.

From the analysis of $N_{f}$ results, it can be seen that in the right part, the arithmetic mean values of $N_{f}$ reach the values corresponding to the left part after the first iteration of the outer loop only after approximately the number of iterations that lead the current temperature to the values of the found minimum ($T_{0}=$20,000 … 40,000).

The search time for the target S-box also changes. Starting from small values of $T_{0}$ with a gradual increase, the search time decreases and at the end of the middle stage can be 1.5–2 times less than the value. Then, given the significant amount of non-productive degradation, the search time increases significantly. The higher the value of $T_{0}$, the greater the amount of nonproductive degradation, and the higher the value of α, the longer it lasts.

If the initial temperature is high or the rate of its decrease is low, a significant number of external cycles is needed to stabilize the system in some local minimum. If the maximum number of external cycles is insufficient, the algorithm may not find a local minimum, which leads to a significant decrease in the number of solutions found or to their complete absence.

The initial temperature at which the probability of finding the target S-box is maximal and no unproductive iterations are observed will be called the *optimal temperature* (labeled as $T_{0}^{\mathrm{opt}}$). The found minimum of the average time of formation of the target S-box corresponds to the initial temperature interval $T_{0}^{\mathrm{opt}}=$20,000 … 40,000. As the parameter α increases, the minimum shifts toward a smaller value of $T_{0}$.

**Figure 22:**
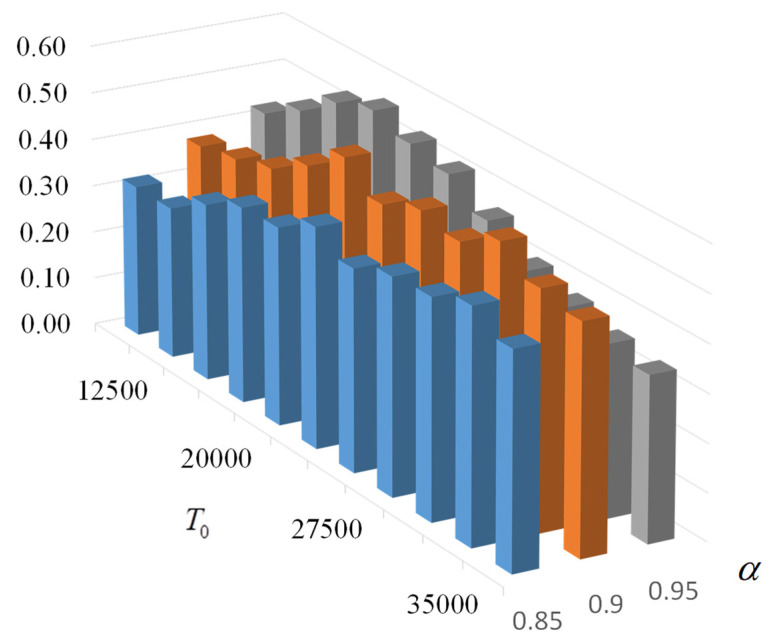
Probability of the target S-box generation when α= 0.85; 0.9; 0.95 and $k_{int}$ = 650 (each point is the average of 1000 tests).

**Figure 23:**
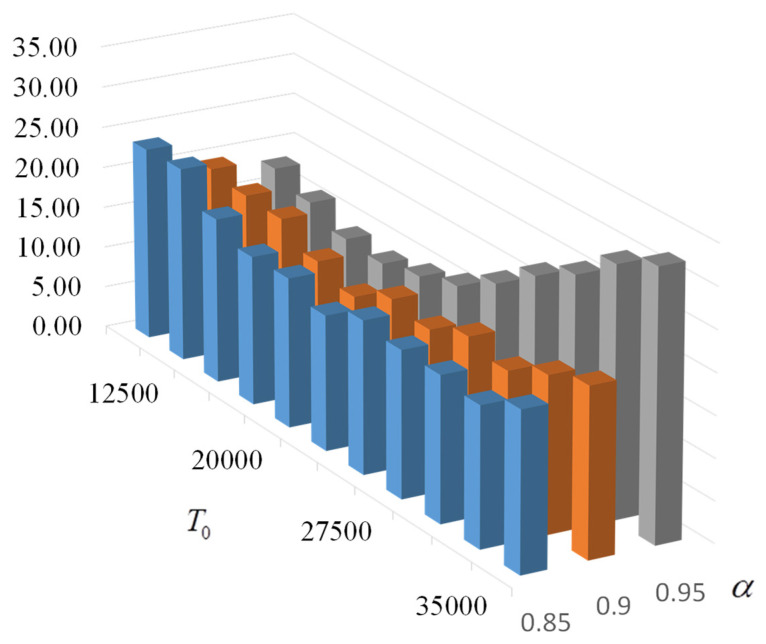
Average time (s) of the target S-box generation when α= 0.85; 0.9; 0.95 and $k_{int}$ = 650 (each point is the average of 1000 tests).

To increase the accuracy of the values obtained, the number of runs for each temperature was increased to 1000. For an acceptable test time, the range of values of $T_{0}=12,500-37,500$ was reduced, and only 11 values of $T_{0}$ were tested with three values of α=0.85;0.9;0.95 (testing was performed for 77 h). The results of the probability of formation of the target S-box and the average time of formation are shown in Figure 22 and Figure 23.

According to the given data, with the chosen parameters ($k_{out}$ = 50, $k_{int}$ = 650, $k_{froz}$ = 5, $K_{THREAD}$ = 30), the best results are obtained at α=0.95 and $T_{0}=20,000$. The probability of finding the target S-box (from $N_{f}=104$) is 56.4%, and the average search time is 14.2 s.

To compare the results with other known implementations of the SA algorithm, Table 2 gives estimates of the difficulty of finding the target S-box (with nonlinearity $N_{f}$). The “-” marks in Table 2 indicate cases with indeterminate indicators.

## 7. Conclusions

We were able to significantly reduce the computational complexity of substitution generation using SA. In addition, we have also significantly increased the probability of generating the target S-boxes with a nonlinearity score of 104.

Based on the results of our studies, we conclude that the simulated annealing method does a good job of finding the target (i.e., with specified properties) S-box. If the algorithm parameters are well chosen, the probability of finding an S-box with nonlinearity $N_{f}=104$ T will be almost unity.

However, a 100% probability of finding the target S-box is not the optimal path in terms of time spent searching. Introducing additional constraints reduces the time spent on each attempt but also reduces the probability of finding the target S-box in each attempt. Therefore, the search results using the simulated annealing method are very sensitive to all input search parameters, and their optimization is a very time-consuming process. The influence of input parameters of simulated annealing method on the search result of target S-box was investigated. Based on the results of the study, the comparative characteristics of the search time and the internal states of the algorithm are presented, and optimization by the search time minimization criterion was carried out. With the chosen algorithm parameters ($k_{out}$ = 50, $k_{int}$ = 650, $k_{froz}$ = 5, $K_{THREAD}$ = 30), the best results were obtained with α=0.95 and $T_{0}=20,000$. In this case, the probability of finding the target S-box (from $N_{f}=104$) is 56.4%, and the average search time is 14.2 s. The algorithm requires about 450,000 search iterations on average. As the number of internal iterations increases, the probability of detecting the target S-box increases to 97%. This is the best known result of applying the SA algorithm to generate bijective 8-bit S-boxes.

## Figures and Tables

**Table 1:** Distribution of the number of search algorithm runs for which no improvement of the cost function was found.

α	The Number of External Loops of the Algorithm for Which No Improvement of the Cost Function Was Found											
	0		1		2		3		4		5	
0.6	4281	42%	3042	30%	1509	15%	725	7%	402	4%	141	1.4%
0.7	2722	36%	2510	33%	1245	16%	641	8%	355	5%	127	1.7%
0.8	1362	24%	2060	36%	1244	22%	592	10%	322	6%	120	2.1%
0.9	2168	26%	1671	20%	1634	20%	1466	18%	955	12%	306	3.7%
0.95	2299	28%	1684	21%	1333	16%	1163	14%	1167	14%	554	6.8%

**Table 2:** Comparison of the results obtained on the generation of bijective 8-bit S-boxes (for different implementations of the SA algorithm).

	SA [11], SA [37]	SA [14]	SA [12]	Our Work
The highest value of $N_{f}$ obtained in the found S-box	102	92	104	104
S-box generation probability	1/200 = 0.5%	-	-	56.4%
S-box generation (search) time	-	-	-	14.2 s
Generation complexity (number of search iterations)	-	-	3,000,000 30,000,000	450,000

## Data Availability

Not applicable.
